# AIF1: Function and Connection with Inflammatory Diseases

**DOI:** 10.3390/biology12050694

**Published:** 2023-05-09

**Authors:** Diego De Leon-Oliva, Cielo Garcia-Montero, Oscar Fraile-Martinez, Diego Liviu Boaru, Luis García-Puente, Antonio Rios-Parra, Maria J. Garrido-Gil, Carlos Casanova-Martín, Natalio García-Honduvilla, Julia Bujan, Luis G. Guijarro, Melchor Alvarez-Mon, Miguel A. Ortega

**Affiliations:** 1 Department of Medicine and Medical Specialities, Faculty of Medicine and Health Sciences, University of Alcalá, 28801 Alcala de Henares, Spain; 2 Ramón y Cajal Institute of Sanitary Research (IRYCIS), 28034 Madrid, Spain; 3 Cancer Registry and Pathology Department, Principe de Asturias University Hospital, 28806 Alcala de Henares, Spain; 4 Unit of Biochemistry and Molecular Biology, Department of System Biology (CIBEREHD), University of Alcalá, 28801 Alcala de Henares, Spain; 5 Immune System Diseases-Rheumatology, Oncology Service an Internal Medicine (CIBEREHD), University Hospital Príncipe de Asturias, 28806 Alcala de Henares, Spain

**Keywords:** allograft inflammatory factor 1, macrophages, microglia, phagocytosis, intracellular signaling

## Abstract

**Simple Summary:**

Allograft inflammatory factor 1 (AIF1) is a calcium-binding protein that participates in intracellular processes such as phagocytosis, membrane ruffling and F-actin remodeling. Due to its multiple functions, AIF1 is linked with the activation of macrophages and several diseases such as kidney disease, rheumatoid arthritis, cancer, cardiovascular diseases, metabolic diseases, neurological disorders and transplants. In this review, we present a comprehensive review of the known structure, functions and role of AIF1 in inflammatory diseases.

**Abstract:**

Macrophages are a type of immune cell distributed throughout all tissues of an organism. Allograft inflammatory factor 1 (AIF1) is a calcium-binding protein linked to the activation of macrophages. AIF1 is a key intracellular signaling molecule that participates in phagocytosis, membrane ruffling and F-actin polymerization. Moreover, it has several cell type-specific functions. AIF1 plays important roles in the development of several diseases: kidney disease, rheumatoid arthritis, cancer, cardiovascular diseases, metabolic diseases and neurological disorders, and in transplants. In this review, we present a comprehensive review of the known structure, functions and role of AIF1 in inflammatory diseases.

## 1. Introduction

The immune system is a collection of cells that protect the respiratory passages, skin, intestinal tract, and other areas from external and internal threats, such as fungi, bacteria, parasites, virus infections, toxins and cancer cells, through complex signaling pathways [1]. The immune system is classified into two types: innate immunity and adaptive immunity. The former is the main line of defense against an introduced pathogen. It is a nonspecific mechanism that is rapidly activated within hours of exposure to a foreign antigen. The latter is antigen-specific and antigen-dependent, and there is a longer period between antigen exposure and the immune response. These two types of immunity are not exclusive processes of host defense, but are often reciprocal, with defects in each system resulting in host vulnerability and insufficient protection [2,3].

Inflammation is the immune system response to damaged cells, pathogens, toxic compounds or irradiation, and its principal aim is to remove harmful stimuli and promote healing [4,5]. Inflammation can occur as a result of both infectious and noninfectious pathologies. At the tissue level, inflammation causes redness, pain, heat, swelling and a reduction in tissue activity, which are produced by local immune, inflammatory and vascular cell responses to injury or infection [6]. During inflammation, important microcirculatory events take place, such as vascular permeability changes, inflammatory mediator release and leukocyte recruitment [7]. If acute inflammation becomes uncontrolled, it can become chronic and lead to the development of chronic inflammatory diseases [8].

Macrophages are immune cells distributed throughout all tissues of the organism, where they maintain homeostasis by accomplishing a great variety of functions, such as metabolic functions, clearance of cellular debris, and tissue remodeling and defense [9]. Tissue-resident macrophages can arise from three different sources: the yolk sac, the fetal liver and blood monocytes. Depending on the localization, these cells acquire a specific phenotype to carry out specific functions, e.g., osteoclasts in the bone, Kupffer cells in the liver, alveolar macrophages in the lungs or Langerhans cells in the skin [10,11]. Macrophages act as sentinel cells in tissues and respond to external stimuli, especially inflammation. The activation of macrophages leads to the formation of two distinct phenotypes: classical M1 and alternative M2. The former is involved in inflammatory responses, and the latter is involved in anti-inflammatory responses, such as tissue repair [12,13]. Macrophages have great motility and secrete pro/anti-inflammatory cytokines, growth factors and phagocyte antigens, which contribute to their role as antigen-presenting cells (APCs) [14,15].

Microglia are resident macrophages localized throughout the central nervous system (CNS), together with neurons, astrocytes and oligodendrocytes [16]. Microglia contribute to CNS homeostasis and accomplish a variety of functions, including neurogenesis, synaptic remodeling and removal of cell debris [17,18]. Additionally, microglial activation is a key player in the response to CNS disorders such as Alzheimer’s disease (AD), ischemic stroke, or multiple sclerosis [19,20]. Microglia arise predominantly from yolk sac-derived macrophages [21]. The morphological characteristics of microglia transition from a ramified structure in the “resting” state to an amoeboid structure in the “activated” state [22]. Moreover, microglia can polarize into classical (M1) or alternative (M2) phenotypes. The former induce inflammation by secreting inflammatory cytokines, while the latter present anti-inflammatory properties, and there is a continuum of intermediate phenotypes [23].

Allograft inflammatory factor 1 (AIF1) is a 17 kDa cytosolic protein that binds calcium and actin, and its expression is induced by cytokines such as IFN-γ [24]. It was originally isolated from infiltrating macrophages in rat cardiac allografts with chronic rejection [25]. The AIF1 gene is encoded within the major histocompatibility complex (MHC) class III region on chromosome 6p21.33, where it is surrounded by surface glycoprotein and complement cascade protein genes, as well as genes involved in inflammation, such as tumor necrosis factor-α (TNF-α), TNF-β or NF-κB genes [26,27]. AIF1 is well conserved across the animal kingdom; it has been described in invertebrates [28], including marine sponges [29] and oysters [30]. Several subsequent studies have linked AIF1 with inflammation [31], immunoinflammatory diseases such as kidney disease [32], rheumatoid arthritis [33], cancer [34] or cardiovascular disease [35] and transplant rejection [36]. Moreover, AIF1 may promote macrophage activation and the growth of vascular smooth muscle cells and T-lymphocytes [31,37,38]. Therefore, AIF1 is a protein that can function as a biomarker and therapeutic target. AIF1 expression seems to be cell type-specific, and it is upregulated under distinct inflammatory stimuli [31]. IFN-γ, TNF-α, interleukin-1β (IL-1β) or T-cell conditioned media are some upregulators of AIF1 expression in macrophages.

In this review, we will focus on the diverse functions of AIF1, especially in intracellular signaling pathways and the activation of macrophages, as well as its connection with diverse inflammatory maladies.

## 2. Structure

**Figure 1:**
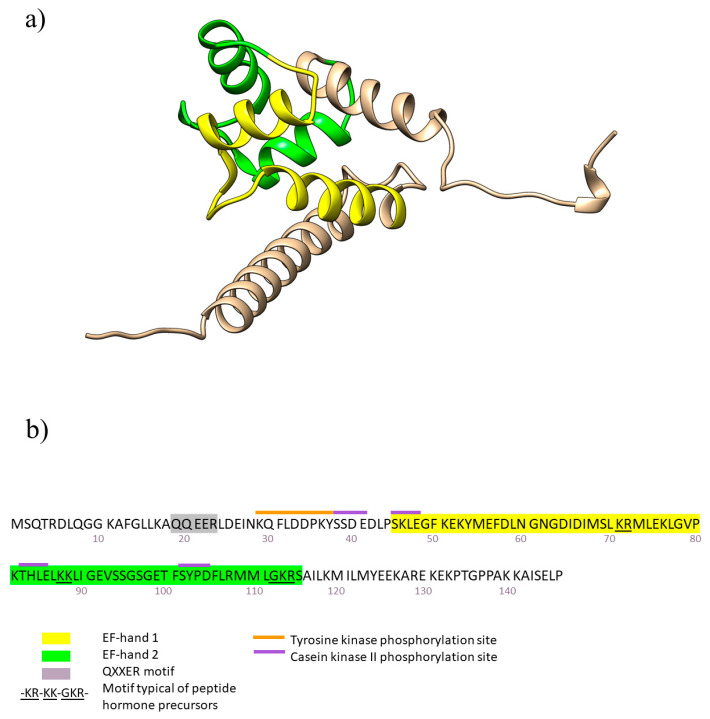
Structure and sequence of AIF1. (**a**) Secondary structure of AIF1. EF-hand 1 and -2 are colored in yellow and green, respectively. AlphaFold code AF-P55008-F1. Illustrated by the program UCSF Chimera. (**b**) 147 amino acid sequence of AIF1. Biologically active sites are labeled as shown in the legend.

AIF1 is a monomeric protein of 147 amino acids. It belongs to the family of EF-hand proteins, which includes other calcium-binding proteins such as troponin C and calmodulin [39]. The EF-hand motif consists of a helix-loop-helix motif in which calcium binds to a loop of 12 residues and induces a conformational change from closed to open (see Figure 1a) [40]. AIF1 possesses two EF-hands, but calcium only binds to the second one, and activates a unique molecular switch different from other classical EF-hand proteins [41]. Moreover, AIF1 also contains an internal 44-residue segment with the sequence pattern –KR–KK–GKR–, a motif typical of peptide hormone precursors [42], several postsynaptic density-95/discs-large/zonula occludens-1 (PDZ) interaction domains, which participate in intracellular signaling complexes [43], a QXXER motif, shown to be critical for regulation by G protein βγ subunits [44], a tyrosine kinase phosphorylation site and a casein kinase II phosphorylation site [45] and a serine-threonine protein kinase that phosphorylates a wide range of substrates involved in cell cycle regulation (see Figure 1b) [46].

The AIF1 homologs ionized calcium binding adaptor molecule 1 (Iba1), microglial response factor (MRF-1) and daintain, presenting identical sequences and only species-specific amino acid differences; therefore, they also present calcium binding activity [24]. As many articles have mentioned, AIF1 is also known as Iba1, MRF-1 and daintain, and for the current purpose of this review, we have only used the term AIF1. There are also alternatively transcribed and spliced mRNAs encoded by the *AIF1* gene: interferon-responsive transcript (IRT-1), G1, balloon angioplasty-responsive transcript (BART-1) and Hara-1 [24]. They present identical coding DNA (cDNA) sequences but have splice-specific differences in the promoter region. However, their functional differences have not been uncovered.

## 3. Functions

### 3.1. Membrane Ruffling

Membrane ruffling, also known as cell ruffling, is the formation of actin membrane protrusions. This process takes place in the cellular zones undergoing fast reorganization of the plasma membrane, and often occurs before the formation of lamellipodia [47]. Ohsawa et al. demonstrated the role of AIF1 in macrophages/microglia by analyzing its intracellular location in the microglial cell line MG5. In the absence of macrophage colony-stimulating factor (M-CSF), AIF1 is present in the cytoplasm, but in the presence of M-CSF, AIF1 translocates to membrane ruffles and colocalizes with F-actin, suggesting that AIF1 contributes to the formation and regulation of membrane ruffles by collaborating with actin [48]. Additionally, this study explains the participation of AIF1 in the formation of phagocytic cups in macrophages, suggesting its contribution to the advanced steps of phagocytosis.

The Rho family GTPases Cdc42, Rho and Rac are recognized as molecular switches that coordinate the remodeling of the actin cytoskeleton [49]. In fibroblasts, Rac is involved in the formation of lamellilopodia and membrane ruffles [50]. Although studies in the MG5 microglial cell line show colocalization of AIF1 with Rac in membrane ruffles, they could not demonstrate that AIF1 binds directly to Rac [48]. Moreover, AIF1 participates in Rac signaling but not in Cdc42 or Rho signaling. Studies by Kazawa et al. showed that phospholipase C-γ (PLC-γ) activates Rac and triggers membrane ruffling in Swiss 3T3 fibroblasts, porcine aorta endothelial (PAE) cells, and Chinese hamster ovary (CHO) cells transfected with AIF1 cDNA [51]. This novel signaling pathway in membrane ruffling could be complementary to the phosphatidylinositol 3-kinase (PI3K) signaling pathway reported in Swiss 3T3 cells. Indeed, some studies have proposed crosstalk between the PI3K and PLC-γ pathways [52,53,54].

### 3.2. F-Actin Binding Activity

AIF1 possesses F-actin binding activity [55,56,57]. In cultured vascular smooth muscle cells (VSMCs), AIF1 binds to and polymerizes F-actin, as investigated by differential sedimentation, and upon stimulation with platelet-derived growth factor (PDGF), AIF1 translocates to lamellipodia, and its interaction with F-actin is significantly reduced [55]. AIF1 shows calcium-sensitive F-actin binding activity because the linkage between the AIF1 EF-hand and calcium is weak, leading to a conformational change rather than sequestration of calcium ions. The polymerization and arrangement of actin in a particular component of the cell is established by the activity of its associated binding proteins. Similar results were obtained by Sasaki et al. in the MG5 cell line, where AIF1 also displays actin binding and crosslinking activity, as assessed by centrifugation assay and electron microscopic examination [56]. Lastly, Ohsawa et al. found in the MG5 cell line that AIF1 binds directly to L-fimbrin, a 68-kDa actin cross-linking protein, and enhances its actin-bundling activity [57]. In vitro, L-fimbrin coimmunoprecipitated with AIF-1 in cell lysates, and proved that endogenous AIF-1 and L-fimbrin colocalized in membrane ruffles induced by M-CSF. AIF1 and L-fimbrin did not affect each other’s F-actin-binding activity, meaning that AIF-1 and L-fimbrin do not competitively bind to F-actin. The complex made by AIF-1 and L-fimbrin potentially increases the stability of the F-actin linkage that controls and drives membrane ruffling and phagocytosis. Perhaps the union of AIF-1 with L-fimbrin promotes a conformational change in L-fimbrin that facilitates the binding action of this protein. It is plausible that the interaction between AIF-1 and L-fimbrin enables the signaling cascades induced by cell adhesion, which in turn increases actin remodeling in microglia/macrophages.

### 3.3. Regulation of Cell Cycle Progression

Cell cycle progression occurs due to a highly organized process that regulates the expression of specific cell cycle proteins [58]. Autieri et al. discovered that transfection of AIF1 cDNA leads to enhanced proliferative capacity in cultured primary VSMCs, which is positively correlated with the expression levels of AIF1, probably due to its ability to bind calcium [59,60]. The experimental results showed a shortening of the cell cycle and aberrant expression of cell cycle proteins cyclins D1, E and B and proliferating cell nuclear antigen (PCNA). Moreover, AIF1 expression promoted VSMC growth, even in the absence of serum growth factors. Subsequent studies showed that enhancement of growth in AIF1-expressing VSMCs was due to autocrine production of the cytokine granulocyte-colony stimulating factor (G-CSF) [61]. G-CSF is secreted into the medium in cultured VSMCs, and it is also a chemoattractant for monocytes. Finally, the authors proposed that AIF1 initiates Rac1 activation and upregulates the expression of G-CSF, leading to a proliferation of VSMCs.

### 3.4. Functions in Immunity: Macrophages, T Lymphocytes and Dendritic Cells

AIF1 has been demonstrated to play a key role in the development of immune responses and inflammatory pathological processes. Immunohistochemical analysis of AIF1 in a normal murine model revealed the expression of AIF1 in all subpopulations of macrophages, except alveolar macrophages and spermatids [62]. Focusing on its role in macrophages, AIF1 has been shown to be crucial for their survival and proinflammatory activity [63]. In vitro studies employing the macrophage cell line CRL-2192 proved that AIF1 upregulation promoted inducible nitric oxide synthase (iNOS) expression levels, nitric oxide (NO) production and macrophage cell migration, but AIF1 downregulation diminished iNOS expression levels and NO production and induced apoptosis of macrophages. Interestingly, the disintegrin and metalloproteinase domain 3 (ADAM3) was identified as an upstream regulator of AIF1 expression [63]. In previous experiments, the macrophage cell line RAW 264.7 was transfected with mouse AIF1 cDNA. Then, macrophages were stimulated with bacterial lipopolysaccharide, and those that overexpressed AIF1 showed marked morphological changes and produced significantly large amounts of interleukin (IL)-6, IL-10 and IL-12p40 [64]. Later studies confirmed the role of AIF1 in the stimulation of chemokine production. By using the high-density oligonucleotide microarray technique, they discovered an upregulation in the expression of CC chemokines such as CCL1, CCL2, CCL3, CCL7, and CCL20 in CD14+ peripheral blood mononuclear cells (PBMCs) treated with recombinant human AIF (rhAIF1), and ELISA confirmed that rhAIF1 promoted the secretion of CCL3 and IL-6 [65]. Moreover, the culture medium from rhAIF1-stimulated CD14+ PBMCs enhanced the migration of PBMCs [65]. Finally, Tian et al. also linked AIF1 with macrophage activation. They inhibited AIF1 expression in RAW264.7 cells with small interfering RNA (siRNA), which led to a decrease in proliferation, migration and signal transduction of Akt and the MAPK family kinases p44/42 and p38, initiated by atherogenic stimuli (oxidized low-density lipoproteins) [66].

Further studies are needed to elucidate the function of AIF1 in T lymphocytes. Kelemen et al. overexpressed AIF1 in the T-lymphoblastoid MOLT-4 cell line by retroviral transduction, and found enhancement of T-lymphocyte proliferation and migration and that AIF1 interaction with nonmuscle actin and conditioned media from AIF1-expressing lymphocytes could influence VSMC proliferation [38]. Furthermore, rhAIF1 potentiated T_H1_ cell differentiation and inhibited the Treg response in PBMCs from healthy individuals [67].

Another proposed role of AIF1 in regulating immunity is that it functions as a mediator in the differentiation and function of dendritic cells (DCs). Silencing AIF1 in murine DCs skews the polarization of CD4+ T naïve cells toward T_H1_ and T_H17_ responses in cocultures and expands IL-10-producing and CD25+ Foxp3+ T regulatory subsets, accompanied by decreased levels of IL-2, TNFα and IFN-γ [68]. Additionally, AIF1 inhibition can inhibit antigen-specific CD8+ T-cell activation, limiting CXCR3, IFN-γ and granzyme B expression and proliferation and, in turn, expanding IL-10-producing CD8+ CD122+ PD-1+ regulatory T cells [69]. Moreover, DNA methyltransferase 3a expression in DCs is involved in the regulation of T_H17_/T_reg cells_ polarization through the c-Jun/AIF1 axis, because c-Jun can bind to the promoter of AIF1 [70]. Finally, AIF1 modulates interferon regulatory factor 8 (IRF8) and transcription factor RelB (by interaction with protein kinase C (PKC) to promote NF-κB and MAPK signaling and drive hematopoietic stem cell differentiation into classical DC type 1 (cDC1) subsets and monocyte-derived DC (Mo-DC) under Fms-related tyrosine kinase 3-ligand (Flt3-L) and granulocyte macrophage-colony stimulatory factor (GM-CSF) stimuli, respectively [71].

### 3.5. Activation of Microglia

Furthermore, AIF1, together with cluster of differentiation 68 (CD68) and major histocompatibility complex-II (MHC-II), is a specific marker of microglial cells in the CNS [72,73,74]. Indeed, it has been suggested that AIF1 upregulation plays a role in microglial activation, through its ability to rearrange the membrane cytoskeleton [51,75]. Norden et al. showed AIF1 immunoreactivity and deramified microglia in mice 24–48 h after lipopolysaccharide (LPS) injection, which corresponded to the resolution phase of activation [76]. Moreover, Lituma et al. developed a murine model *AIF1* -/-, in which microglia displayed reductions in ATP-induced motility and ramification, reduced excitatory synaptic connections and, in adult mice, behavioral alterations [77]. Finally, AIF1 is upregulated in response to programmed neuronal cell death and degeneration, implying a functional role of MRF-1-expressing microglia [78].

### 3.6. Transcytosis in M Cells

**Figure 2:**
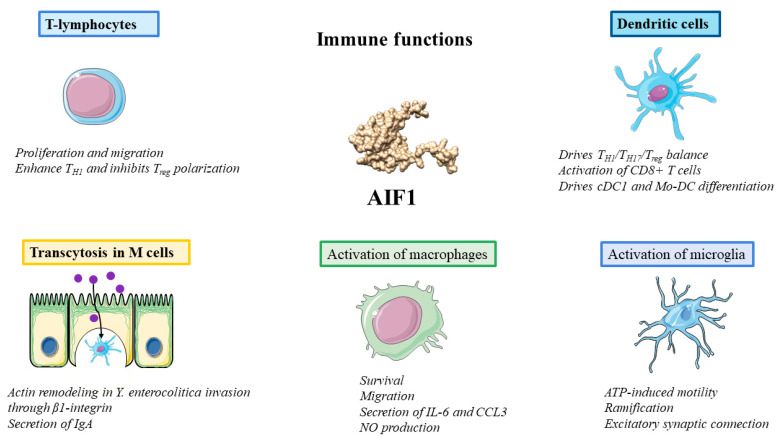
Overview of immune functions: activation of macrophages, activation of microglia, polarization of T-lymphocytes and dendritic cells and transcytosis in M cells. IL-6: interleukin-6, CCL3: C-C Motif Chemokine Ligand 3, NO: nitric oxide, ATP: adenosine triphosphate, IgA: immunoglobulin A, DNMT3: DNA methyltransferase, IRF8: interferon regulatory factor 8, cDC1: classical dendritic cell type 1, Mo-DC: monocyte-derived dendritic cell.

Microfold (M) cells are specialized phagocytic epithelial cells that uptake antigens from the intestinal lumen and deliver them to dendritic cells for antigen presentation [79]. The antigens are transported through M cells by the transcytosis process [80]. Kishikawa et al. described a novel role of AIF1 in M cell transcytosis. They developed *AIF1* -/- model mice that demonstrated suppressed uptake of particles and commensal bacteria, even though M cell development and morphology were unaffected [81]. More specifically, AIF1 was associated specifically with actin remodeling during *Yersinia enterocolitica* invasion. However, they could not link the absence of AIF1 with membrane ruffling, which is induced by invasion of *Salmonella enterica* serovar Typhimurium [82]. *Y. enterocolitica* invasion leads to the generation of secretory immunoglobulin A (sIgA) antibodies [83], which was also diminished in AIF-deficient mice, so AIF1 may also contribute to the mucosal immune system. It appeared that *Y. enterocolitica* invasion occurred through the activation of β1 integrin via AIF1 [81]. However, AIF1-independent uptake of some commensal bacteria occurs continuously through M cells. Finally, AIF1 is also expressed in the podocytes of kidneys where, although its function is yet to be uncovered, it may be involved in the transport of materials, similar to that in M cells (see Figure 2) [84].

### 3.7. Activation of Endothelial Cells (ECs)

**Figure 3:**
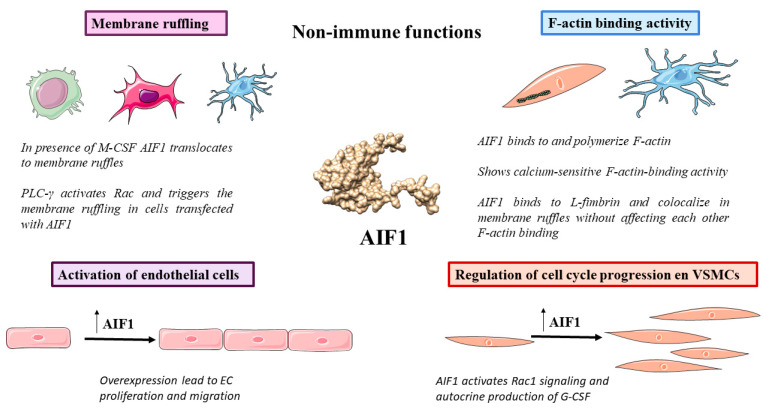
Overview of nonimmune functions of AIF1: membrane ruffling, F-actin binding-activity, activation of endothelial cells and regulation of cell cycle progression. M-CSF: macrophage-colony stimulating factor, AIF1: allograft inflammatory factor 1, PLC-γ: phospholipase C-γ, EC: endothelial cell, VSMC: vascular smooth muscle cell, G-CSF: granulocyte-colony stimulating factor.

Finally, AIF1 has also been related to the activation of ECs. Overexpression of AIF1 in a human umbilical vein EC line (HUVEC) by transfection of the vector pcDNA3.1(-) promoted their proliferation and migration [85]. In contrast, the inhibition of AIF1 expression in primary bovine aortic endothelial cells (BAECs) by the transfection of AIF1 siRNA significantly reduced EC proliferation and migration and the activation of mitogen-activated protein kinase p44/42 and PAK1 (signal transduction kinases) [86]. Additionally, overexpression of AIF1 in aortic rings promoted EC tube-like structure and microvessel formation from mouse thoracic aortic rings [86]. Overall, these data show that AIF1 mediates EC activation and new vessel formation (see Figure 3).

## 4. Clinical Relevance

We have reviewed the variety of cell-specific functions displayed by AIF1. Next, we explored the role of AIF1 in disease. Relevant studies have linked AIF1 with inflammatory diseases such as kidney disease, rheumatoid arthritis, cancer, cardiovascular diseases (CVDs), neurological disorders and transplants. Interestingly, our group has published the fact that serum AIF1 levels decline during anti-TNF treatment in patients with inflammatory bowel disease and Crohn’s disease [87,88]. In this section, the main implications of AIF1 action in these maladies are presented (see Table 1).

### 4.1. Kidney Disease

Several studies have linked AIF1 to chronic kidney disease (CKD) and diabetic kidney disease (DKD). CKD is a general term for heterogeneous disorders that affect the structure and function of the kidney, and this damage is present for 3 months or more [89]. CKD patients have a higher risk of developing CVD, and vascular calcification (VC) is one of the strongest predictors of CVD risk [90]. Serum aldosterone levels are increased in CKD patients, and mediate the VC process [91,92]. VC is a highly regulated process in which calcium-phosphate complexes are deposited in the intima and media and participate in ECs, VSMCs, circulating cells and pro-osteogenic and anti-calcifying factors [93,94]. According to Chang et al., aldosterone induced VC through the AIF1 signaling pathway [32]. By coculturing ECs and VSMCs, we found that aldosterone upregulated AIF1 in ECs, leading to calcium influx and calcification of VSMCs. Additionally, in a mouse model, the aldosterone inhibitor spironolactone downregulated the expression of AIF1, and an AIF1 knockout model of CKD presented attenuated calcification of the aortic wall [32]. Thus, further research on the AIF1-triggered mechanisms in ECs and intercommunication with VSMCs is needed to find proper therapeutic targets that block or reduce VC in CKD patients. Finally, uremia is the accumulation of uremic solutes in plasma, and develops during CKD [95,96]. VC also occurs during uremia, and Hao et al. demonstrated that AIF1 mediates the crosstalk between calcium ions and aldosterone and regulates NF-κB activity in the inflammation and calcification of VSMCs during uremia [97].

DKD is classified as a microvascular complication of diabetes, and is initiated as a result of the dysregulated metabolic milieu: hyperglycemia, hyperlipidemia, and insulin resistance [98,99]. In addition, several studies have linked DKD with inflammation and oxidative stress [100,101,102,103]. Recent results from Fu et al. identified AIF1 as the mediator of glomerular endothelial cell inflammation and oxidative stress in DKD via the NF-κB signaling pathway [104]. Indeed, downregulation of AIF1 reversed pathological damage, renal inflammation, and oxidative stress in a DKD murine model. AIF1 has also been linked to inflammation, oxidative stress and autophagy via the miR-34a/autophagy-related gene 4B (ATG4B) pathway [105]. miR-34a targets the 3′ untranslated region of ATG4B mRNA and inhibits its translation [106,107]. In human renal glomerular endothelial cell (HRGEC) culture, AIF1 overexpression or silencing upregulates or downregulates miR-34a and downregulates or upregulates ATG4B, respectively [105]. Additionally, glucose induced the expression of AIF-1, miR-34a and reactive oxygen species (ROS) and inhibited ATG4B expression. Blood and urine samples from DKD patients and murine models showed high levels of AIF1, miR-34a, oxidative stress and inflammatory factors [105]. Finally, serum AIF1 levels were measured in 248 type 2 diabetes patients and were positively correlated with albuminuria and negatively correlated with the estimated glomerular filtration rate (eGFR) [108]. Authors suggested that levels of AIF1 could be used as markers of macrophage activation, which induces renal injury and leads to the development of DKD.

Finally, renal interstitial fibrosis (RIF) is the last common step in all CKD pathologies. Chronic inflammation in response to renal injury triggers the overdeposition and accumulation of extracellular matrix (ECM), collagen, and related molecules in the renal interstitium, by myofibroblasts [109]. Macrophages play a key role in RIF, including the secretion of inflammatory and anti-inflammatory cytokines, as mediators of tissue repair such as transforming growth factor-β, Wingless and Int-1 (Wnt) ligands, PDGF, connective tissue growth factor (CTGF) and components of the renin-angiotensin system, the recruitment/proliferation/activation of fibroblasts, and macrophage–myofibroblast transition [110,111,112]. To uncover the mechanisms through which macrophages drive fibrosis, Li et al. showed AIF1 upregulation in macrophages in the renal interstitium of an RIF animal model [113]. Next, the RAW 264.7 cell line was stimulated with aldosterone, leading to the upregulation and secretion of AIF1. RAW 264.7-aldosterone-activated cells were cocultured with fibroblasts, and after 72 h the levels of α-smooth muscle actin (α-SMA), a marker of myofibroblasts, and phosphorylated p38 (p-p38) in fibroblasts were upregulated, indicating that AIF1 promotes RIF via p38. However, subsequent studies have shown that AIF1 upregulation induced by aldosterone may act through AKT/mTOR oxidative stress pathways in macrophages [114].

### 4.2. Rheumatoid Arthritis

Rheumatoid arthritis (RA) is a chronic inflammatory autoimmune disease with extensive degradation of the cartilage in joints, and is characterized by autoantibodies against rheumatoid factor (RF, anti-immunoglobulin G) and anti-citrullinated protein antibodies (ACPAs) [115,116,117]. Kimura et al. were the first to evaluate the role of AIF1 in RA. They found that AIF-1 was strongly expressed in the infiltrating mononuclear cells and synovial fibroblasts of RA patients, compared with osteoarthritis (OA) patients. Moreover, the levels of AIF1 in synovial fluid were also higher, and AIF1 induced the proliferation and expression of interleukin-6 (IL-6) in cultured synovial cells [118]. In addition, Harney et al. performed immunohistochemical analyses on the synovium from RA patients, and showed that AIF1-expressing cells were synovial macrophages, while there was little or no expression of AIF1 in synovium from OA patients [119]. Further studies from Pawlik et al. showed that the peripheral blood of RA patients presents increased numbers of circulating AIF1-expressing monocytes, and is positively correlated with the DAS28 score and the Sharp erosion score [120]. Piotrwska et al. also found an increased number of AIF1-expressing cells in blood and synovial membranes in RA patients, compared to OA patients [33]. Collectively, these results indicated that AIF1 plays a role in the pathogenesis of RA, probably as an inflammatory factor that activates macrophages, and its expression could be upregulated by other proinflammatory factors.

Finally, there are studies that link *AIF1* gene polymorphisms with RA. These results suggest that the *AIF1* rs2259571 CC genotype is associated with the active form of RA and poor response to therapy with methotrexate (MTX) [121,122]. Therefore, it could be useful to screen for this polymorphism at the time of RA diagnosis, to personalize treatment. However, the specific role of the *AIF1* rs2259571 CC genotype in RA pathogenesis and MTX resistance is not yet understood.

### 4.3. Cancer

Cancer refers to a group of diseases in which abnormal cells acquire the capability of uncontrolled and unlimited proliferation, self-sufficient growth, tissue invasion and metastasis [123]. Moreover, inflammation is a hallmark of cancer that promotes tumorigenesis and impairs immune surveillance and response to treatment [124,125,126]. Innate and adaptative immune responses are triggered in response to tumor cells [127,128]. Tumor-associated macrophages (TAMs) can polarize either to the M1-antitumoral phenotype driven by IFN-γ or the M2-protumoral phenotype driven by IL-4 or IL-13 [129,130]. As a result, several studies have linked the expression and function of AIF1 to the development of cancer, with the best-studied cancer types being hepatocellular carcinoma (HCC), breast cancer and glioma.

In relation to HCC, AIF1 enhances proliferation of the human hepatoma cell line HepG2 through activation of the insulin growth factor/insulin-like growth factor-1 receptor (IGF/IGF1R) axis and its downstream signaling pathway [131]. Moreover, the expression of AIF1 was upregulated in HCC tissue compared to adjacent healthy tissue, and correlated positively with the following clinicopathological characteristics: median tumor size, number of tumor deposits, Barcelona Clinic Liver Cancer stage and portal vein tumor thrombus [132]. Additionally, the silencing of AIF1 in the liver cancer cell lines Huh7 and SMMC7721 inhibited proliferation. Thus, AIF1 could be used as a biomarker and therapeutic target.

Several studies have linked different roles of AIF1 with the development and pathogenesis of breast cancer. Immunohistochemistry analyses revealed high expression of AIF1 in the epithelia of patients with ductal carcinoma [133]. Furthermore, overexpression or silencing of AIF1 in the breast cancer cell line MDA-MB-231 led to enhanced and decreased proliferation, respectively. Using a luciferase reporter assay, the authors discovered that AIF1 activates NF-κB signaling and upregulates the expression of cyclin D1 [133]. Subsequent studies also elucidated the fact that AIF1 promotes breast cancer cell migration through the activation of the p38 MAPK signaling pathway, which upregulates the expression of tumor necrosis factor-α (TNF-α) [134]. These experiments were performed with MDA-MB-231 and MCF-7 cells in macrophage-conditioned medium with or without AIF1. A recent study from Slim et al. identified two differentially expressed *AIF1* isoforms in families with a high risk of breast cancer and no deleterious BRCA1/BRCA2 mutations [45]. Interestingly, the *AIF1v1* isoform was mostly expressed in infiltrating lymphocytes, while the *AIF1v3* isoform was highly expressed in infiltrating macrophages in breast adipose tissue. The number of infiltrated lymphocytes positively correlated with *AIF1v1* expression in the adipose tissue, and *AIF1v1* expression was correlated with age, menopausal status, and Cytochrome P450 Family 19 Subfamily A Member 1 (CYP19A1) and IL-6 expression [45]. Another study used a mouse model of breast cancer metastases, and identified AIF1 upregulation in the metastasis-associated macrophages (MAMs) of metastatic breast cancer cells [135]. However, AIF1 deficiency in MAMs did not affect the number of lung metastases; therefore, the role of AIF1 in the metastasis of breast cancer cells seems to be limited to the activation of MAMs. Finally, AIF1 appears to participate in the resistance of breast cancer cells to cisplatin by enhancing the uptake of intracellular cisplatin out of the cell and inhibiting apoptosis via the activation of Bcl-2 and suppression of p53 and Bax [136].

In patients with brain glioma, including low-grade glioma and glioblastoma multiforme, AIF1 expression is upregulated [137]. AIF1 expression was positively correlated with the level of immune infiltration and poor prognosis. An early study in rat C6 glioblastoma and 9 L gliosarcoma tumor models and human astrocytomas found AIF1 expression in a distinct subset of tumor-associated activated macrophages/microglial cells that accumulated in specific areas of the tumor [138].

Finally, AIF1 has also been linked to other cancer types. In patients with esophageal cancer, AIF1 was identified as a prognostic factor, and was associated with the regulation of the infiltration of macrophages, T cells, T regulatory cells and natural killer cells (NK) through T-cell immunoreceptor with Ig and ITIM domains (TIGIT) expression, an inhibitory immune checkpoint [34,139]. With respect to infantile hemangioma, AIF1 expression was found specifically in ECs, and the authors suggested that this EC-specific expression may trigger the recruitment of myeloid cells [140]. In non-small cell lung cancer (NSCLC), AIF1 expression was upregulated, and associated with metastasis, higher TNM stage, and poorer survival [141]. Moreover, AIF1 may promote this aggressive tumor function via the activation of p38-MAPK and JAK/STAT signaling. In a gallbladder cancer mouse model, the inhibition of AIF1 leads to the decreased secretion of inflammatory factors and cell proliferation, augments cell apoptosis, and limits the invasion and epithelial-mesenchymal transition (EMT) of cancer cells via the TGF-β1/p38 pathway [142]. Finally, unlike the abovementioned cancers, AIF1 expression in gastric cancer tissue is significantly downregulated when compared to that in normal tissue [143]. AIF1 silencing in gastric cancer BGC-823 and SGC7901 cell lines led to enhanced proliferation and upregulation of β-catenin, a molecule that has been demonstrated to play an important role in gastric cancer [144].

### 4.4. CVDs

Atherosclerosis is a chronic inflammatory disease characterized by the accumulation of lipids in the walls of large and medium arteries, the activation of proinflammatory signaling pathways, the expression of cytokines/chemokines, the recruitment of immune cells, including monocyte-derived macrophages, and excessive ROS production [145,146]. Atherosclerosis increases the risk of heart attacks, ischemic stroke and peripheral vascular disease [147]. Circulating monocytes are recruited to arterial walls in response to the inflammation caused by lipid accumulation, transform into macrophages and start uptaking oxidized lipoproteins, which transform macrophages to a foam cell phenotype [148,149]. Macrophages also contribute to the stabilization of atherosclerotic plaques through tissue remodeling, fibrous cap formation and apoptotic cell clearance [150,151].

AIF1 appears to be involved in the development of atherosclerosis. Mishima et al. used *ApoE* -/- mice, an animal model of human atherosclerosis [152], to generate an *ApoE* -/- *AIF1* transgenic model. This model showed an increased area of atherosclerotic lesions in comparison to that of atherosclerotic lesions in *ApoE* -/- mice [37]. In addition, mouse macrophages transfected with AIF1 and stimulated peritoneal exudate cells from transgenic mice showed increased phagocytic activity of latex beads and *Escherichia coli* BioParticles, as well as incorporation of acetylated low-density lipoprotein (LDL). Immunohistochemical staining of AIF1 in artery segments with atherosclerotic plaques from coronary heart disease patients showed high positive staining of AIF1 in the tunica intima and media, and positive AIF1 staining was not present in the artery segments without atherosclerotic plaques [35]. Moreover, the injection of AIF1 in BALB/c mice leads to increased levels of serum C-reactive protein, a risk factor and indicator of CVD [153], augmented blood oxidative activity, increased oxidation of lipoproteins, and decreased superoxide dismutase activity, which promote oxidative stress [35]. Additionally, U937 macrophages exposed to exogenous AIF1 demonstrated intense uptake of oxidized, low-density lipoprotein (ox-LDL), probably due to an upregulation of scavenger receptor A (SRA). Finally, Egaña-Gorroño et al. extended the known role of AIF1, and showed that high-fat-diet-induced atherosclerotic plaques in *AIF1* -/- *ApoE* -/- mice present larger necrotic cores than those in *ApoE* -/- mice [154]. In addition, primary macrophages from *AIF1* -/- mice displayed increased susceptibility to oxidative stress and lipid overload and possessed impaired phagocytosis and efferocytosis. It was demonstrated that AIF1 activated macrophages through the NF-κB pathway, and is specifically required for phosphorylation of p65 at Ser536. Under stimulation, AIF1 upregulates the expression of pro-inflammatory, anti-apoptotic, and stress response-related genes [154]. In addition to AIF1-expressing macrophages, VSMCs also express AIF1 in atherosclerotic plaques from *ApoE* -/- mice, and VSMCs overexpressing AIF1 upregulated the expression of matrix metalloproteinases-2 and -9 and increased NF-κB pathway activation and uptake of ox-LDL; therefore, VSMCs act similarly to macrophages in atherosclerosis [155]. Finally, Albiero et al. demonstrated that AIF1 plays a role in promoting atherosclerotic calcification, through the paracrine activity of myeloid calcifying cells and overexpression in resident cells [156].

### 4.5. Metabolic Disease

Obesity is linked to low chronic inflammation, and macrophages are recruited to adipose tissue, promoting inflammation and releasing TNF-α, IL-6, and adiponectin [157,158]. Fukui et al. found that serum levels of AIF1 were positively correlated with metabolic parameters such as levels of fasting plasma glucose, hemoglobin A_1C_, triglycerides, uric acid, waist circumference and body mass index, while they were negatively correlated with high-density lipoprotein levels in healthy subjects [159]. In a study of 510 obese people, the single nucleotide polymorphism rs2844479 in the *AIF1* gene was associated with an increased risk of obesity in the Greek population [160].

Lorente-Cebrián et al. were the first to describe AIF1 as an adipokine released by adipose-tissue macrophages [161]. Indeed, AIF1 mRNA levels were higher in obese women than in nonobese women, and AIF1 mRNA levels normalized after reaching a nonobese weight-stable state. Additionally, AIF1 mRNA levels were positively correlated with insulin resistance as evaluated by the homeostasis model assessment for insulin resistance (HOMA) [161]. Subsequent experiments from Ren et al. revealed that the 3T3L1 adipocyte cell line cultured with macrophage-conditioned medium transfected with AIF1 showed a slight intracellular accumulation of lipids, increased production of ROS, elevated release of TNF-α, IL-6 and resistin and decreased secretion of adiponectin [162]. These findings showed that glucose uptake and consumption was suppressed, the NF-κB pathway was activated, GLUT4 expression on the plasma membrane was downregulated, and Akt phosphorylation was reduced [162]. Male mice lacking AIF1 showed relative resistance to hyperglycemia and a high-fat diet, and their adipose-tissue levels of norepinephrine (NE) were higher, due to less monoaminoxidase A activity and NE clearance by macrophages, suggesting that AIF1 silencing protected against obesity [163]. Moreover, patients with obesity and hepatic dysfunction showed a large loss of microglia expressing AIF1 [164]. Collectively, AIF1 secreted by adipose-tissue macrophages is an important crosstalk mediator in the physiology of adipocytes, and AIF1 deregulation is involved in obesity, so it may be a potential therapeutic target for anti-obesity treatments.

Regarding type 1 diabetes, biobreeding (BB) rats showed AIF1-expressing macrophage accumulation during insulitis, affecting pancreatic islets [42]. Moreover, rat insulinoma INS-1 cells treated with AIF1 displayed decreased cell viability and glucose-stimulated insulin secretion and increased cell apoptosis [165]. In nonobese diabetic (NOD) mice, AIF1 serum levels were higher during insulitis, compared to those in healthy mice [166]. Additionally, AIF1 injection led to increased glycemia, impaired insulin expression and accelerated type 1 diabetes. Finally, silencing AIF1 expression in NOD mice led to diminished infiltration of immune cells, effector T cells and DCs and higher expression of insulin and numbers of Treg cells in the pancreas [167].

Diabetic retinopathy (DR) is another microvascular complication of diabetes mellitus, and involves the activation of microglia [168,169]. A pilot study showed that the AIF1 serum levels positively correlated with hyperreflective intraretinal spots (HRS) in patients with both nonproliferative or proliferative DR, and correlated with serum levels of proinflammatory cytokines such as IL-1β, IL-6 or TNF-α [170]. Gu et al. identified AIF1 as a candidate gene that promotes angiogenesis in early DR via the regulation of cell migration [171]. However, the results from Fukui et al. did not investigate serum AIF1 levels in DR model mice [108].

### 4.6. Neurological Disorders

We have reviewed the current knowledge of AIF1 function in the activation of microglia (see Functions). As microglia play a role in the homeostasis of the brain, together with recruited macrophages, they are involved in several diseases [172].

First, Kenkhuis et al. measured three markers, AIF1, transmembrane protein 119 (TMEM119) and purinergic receptor P2Y12, in the microglia of healthy control individuals and Alzheimer’s disease (AD) patients [173]. In the healthy individuals, the majority of microglia expressed all three markers, while most of the microglia from AD patients expressed only AIF1. In another study, the levels of AIF1 did not differ between the sexes in biopsied brains from AD patients, but were associated with age and correlated positively with chitinase-3-like protein 2 (CHI3L2), a glycoprotein secreted by macrophages, dendritic cells, osteoclasts and highly proliferative cells involved in the immune infiltration of gliomas [174,175]. In another study, AIF1 expression was positively correlated with another protein, chitinase domain-containing protein 1 (CHID1), which has been associated with macrophage infiltration in colorectal cancer patients and a favorable prognosis in NSCLC patients [176,177,178]. Finally, a systematic review concluded that, despite the heterogeneous results, AIF1 is a pan-microglial marker that is upregulated in AD patients; however, the number of microglial cells remains unchanged [179].

In human focal cerebral infarction, an increase in microglia in the area of glial reaction was measured during the first three days and lasted until the chronic cystic stages [75]. Moreover, the expression of AIF1 in microglia, macrophages and neurons of patients with Creutzfeldt-Jakob disease (CJD) was upregulated [180]. In addition, inducing oxidative stress by adding H_2_O_2_ to the medium of human SKNSH neuroblastoma cells upregulated and increased the secretion of AIF1 into the medium, but its role remains to be elucidated [180]. Infection with the Borna disease virus in Lewis rats leads to meningoencephalitis. AIF1 was upregulated and associated with the activation of microglia and infiltration of macrophages during inflammation [181]. Furthermore, it was concomitantly upregulated by heme oxygenase-1, an antioxidative stress enzyme thought to initiate a neuroprotective response.

Spinal cord injury induced in rats leads to an accumulation of microglia and macrophages expressing AIF1 in parenchymal pan-necrotic areas and perivascular Virchow–Robin spaces and a late activation of macrophages and microglia expressing AIF1 in zones of delayed neuronal cell death [182]. In addition, treatment with the corticosteroid dexamethasone in traumatic brain injury model rats suppressed the accumulation of AIF1-expressing cells at days 1 and 2 after treatment, and is therefore a rapid but transient treatment [183]. Thus, AIF1 may play a role in the response to CNS injury by regulating the immune response, activating microglia and clearing cellular debris.

AIF1-expressing cells in chronic inflammatory demyelinating polyneuropathy (CIDP) and vasculitic neuropathy (VAS) are macrophages mainly in the endoneurium, T cells near the blood vessels, and VSMCs in vessel walls [184].

Experimental autoimmune neuritis (EAN) is an animal model of Guillain-Barré syndrome, an immune-mediated acute polyradiculoneuropathy normally triggered after gastrointestinal or respiratory infection [185]. AIF1 is upregulated in the spleen and sciatic nerves during the preclinical and clinical stages of EAN, while increased serum AIF1 levels are only observed during the preclinical stage [186]. Another study of experimental autoimmune encephalomyelitis (EAE), neuritis, and uveitis in rats showed that there were AIF1-expressing macrophages within autoimmune lesions and AIF1-expressing microglia in the injured brain [187]. Finally, the inhibition of AIF1 expression in an EAE rat model, which can be used as an animal model for human multiple sclerosis (MS), led to a lower risk of developing EAE, less CNS leukocyte infiltration and demyelination, limited CD4+ T-cell expansion in the spleen and downregulated proinflammatory cytokine expression [188].

### 4.7. Transplants

Utans et al. first isolated AIF1 from the infiltrating macrophages of chronically rejected cardiac allografts in Lewis F344 model rats [25] and afterward in the infiltrating macrophages of heart transplants in human patients, suggesting that AIF1 has a similar function in both species [189]. Autieri et al. examined 157 endomyocardial biopsy specimens from 26 patients, and found that AIF1 expression and the amount of expression correlated with rejection and the severity of rejection, respectively [190]. Indeed, AIF1 expression was associated with the development of cardiac allograft vasculopathy (CAV), which is the immune-mediated loss of cardiac grafts by a long-term response against graft vessels, which elicits VSMC proliferation [191]. Moreover, Zhou et al. showed AIF1 expression in the cardiomyocytes and mononuclear cells of cardiac allografts in other heart diseases [36]. In the case of heart transplants, AIF-1 was associated with the severity of cardiac allograft rejection and Quilty B lesions but not Quilty A lesions.

Moreover, AIF1 expression also plays a role in kidney transplants. In particular, AIF1-expressing macrophages were found in the infiltrate of rejected renal biopsies and in patients with acute renal dysfunction associated with clinical rejection episodes [192]. In addition, AIF1 can also be expressed in the podocytes of both healthy donors and diseased patients [84]. Furthermore, two studies have evaluated *AIF1* gene polymorphisms in kidney transplant recipients. One study found that the nonsynonymous (Arg to Trp) rs2269475 single nucleotide polymorphism was associated with a lower risk of rejection in Hispanic kidney transplant recipients [193]. The other study included 269 white kidney transplant recipients but did not discover any *AIF1* polymorphisms that were associated with long-term kidney allograft function [194].

**Table 1:** Overview of the expression of AIF1 and the connection with disease.

Pathology				AIF1 Expression Level	Expressing-AIF1 Cell Type	Target	Mechanism of Action/Correlation	References
Kidney disease	CKD			Upregulated	EC and VSMCs		Aldosterone upregulates AIF1, leading to calcium influx and calcification of VSMC	[32]
	DKD			Upregulated	Glomerular EC	NF-κB signaling pathway	Inflammation and oxidative stress	[104]
				Overexpression	HRGEC	miR-34a	miR-34a targets ATG4B Glucose upregulates AIF1	[105]
				High levels	Serum		Correlated positively with albuminuria and negatively with eGFR	[108]
	RIF			Upregulated	Macrophages of renal interstitium	p38 or Akt/mTOR	Aldosterone upregulates AIF1, leading to formation of myofibroblasts via p38 in fibroblasts and activates macrophages via Akt/mTOR	[113,114]
Rheumatoid arthritis				Upregulated	Monocytes, infiltrating macrophages, synovial fibroblasts, blood and synovial fluid		Circulating monocytes correlated positively with DAS28 and Sharp erosion score AIF1 rs2259571 CC genotype associated with active form of RA and resistance to MTX	[120,121,122]
Cancer	HCC			Upregulated	HCC	IGF/IGF1R axis	AIF1 activates IGF/IGF1R pathway AIF1 expression correlates with median tumor size, number of tumor deposits, the Barcelona Clinic Liver Cancer and portal vein tumor thrombus	[131,132]
	Breast cancer			Upregulated	Epithelia of ductal carcinoma and MAMs	NF-κB Cyclin D1 p38	Proliferation via NF-κB and upregulating cyclin D1 Migration via p38, which upregulates TNF-α Resistance to cisplatin	[133,134,135,136]
					Lymphocytes and macrophages infiltrated	IL-6	*AIF1v1* expression was correlated with age, menopausal status, and CYP19A1 and IL-6 expression	[45]
	Glioma			Upregulated	Microglia and macrophages		Positively correlated with the level of immune infiltration and poor prognosis	[137,138]
	Esophageal cancer			Upregulated		TIGIT	AIF1 identified as a prognostic factor and associated with infiltration of macrophages, T cells, T_reg_ cells and NK through TIGIT expression	[34,139]
	Haemangioma			Upregulated	EC		Trigger the recruitment of myeloid cells	[140]
	NSCLC			Upregulated		p38 and JAK/STAT	Associated with metastasis, higher TNM stage, and poorer survival via activation of p38-MAPK and JAK/STAT signaling	[141]
	Gallbladder cancer			Upregulated		TGF-β1/p38 pathway	Secretion of inflammatory factors, cell proliferation, inhibition of cell apoptosis and invasion and EMT, via TGF-β1/p38 pathway	[142]
	Gastric cancer			Downregulated		β-catenin	Proliferation and upregulation of β-catenin	[143]
Atherosclerosis				Higher	Macrophages of atherosclerotic plaque in arterial wall	p65 of NF-κB pathway	ApoE -/- AIF1 mice showed increased area of atherosclerotic lesion Macrophages transfected with AIF1 incorporate higher levels of LDL, due to upregulation of SRA AIF1 activates macrophages through NF-κB pathway (phosphorylation of p65)	[35,37,154]
				Higher	VSMCs		VSMCs express MMP2/9 and incorporate LDL	[155]
				Higher	Myeloid calcifying cells		Myeloid calcifying cells promote calcification of atherosclerotic plaque through paracrine activity	[156]
Metabolic diseases			Obesity	Higher	Adipose-tissue macrophages		Macrophages secrete AIF1, increasing intracellular accumulation of lipids, production of ROS and release of TNF-α, IL-6 and resistin, and decreasing secretion of adiponectin; glucose uptake was suppressed and less consumed, NF-κB pathway was activated, with decreased GLUT4 on the plasma membrane and reduced Akt phosphorylation	[162]
				Higher	Serum		Correlate positively with fasting plasma glucose, hemoglobin A1C, triglycerides, uric acid, waist circumference and body mass index	[159]
			Type 1 diabetes	Higher	Macrophages in pancreas		Accumulation in insulitis pancreatic islets	[42]
			DR		Serum		Correlated positively with HRS and IL-1β, IL-6 or TNF-α	[170]
Neurological disorders			AD	Upregulated	Microglia		Microglia loss expression of TMEM119 and P2Y12. Correlated positively with age and CHI3L2 and CHID1	[173,174,176]
			Cerebral infarction	Upregulation	Microglia		Detected in first three days of glial reaction	[75]
			CJD	Upregulation	Microglia, macrophages and neurons			[180]
			Borna disease virus infection	Upregulated	Microglia		Activation of microglia and infiltration of macrophages	[181]
			CNS injury	Upregulated	Microglia and macrophages		Accumulation of microglia and macrophages in parenchymal pan-necrotic areas and perivascular Virchow–Robin spaces. Dexamethasone reduces accumulation of AIF1-expressing cell	[182,183]
			CIDP and VAS	Upregulated	Macrophages, T cells and VSMCs		Macrophages mainly in endoneurium, T cells near the blood vessels and some VSMCs in vessel walls	[184]
			EAN	Upregulation	Spleen and sciatic nerves		Upregulation of AIF1 at preclinical, and at height of clinical, EAN	[186]
			EAE	Upregulated	Macrophages and microglia		Macrophages in autoimmune lesions and microglia in the injured brain Inhibition of AIF1 led to a lower risk of developing EAE	[187]
Transplants		Cardiac		Upregulated	Cardiomyocytes and mononuclear cells		Correlated with rejection and associated with CAV and Quilty B lesions	[36,190,191]
		Kidney		Upregulated	Infiltrating macrophages and podocytes		Acute renal dysfunction associated with clinical rejection rs2269475 SNP associated with a lower risk of rejection	[192,193]

## 5. Conclusions and Future Directions

AIF1 was first isolated from chronically rejected cardiac allografts in rats. Since then, several studies have demonstrated that AIF1 is a key mediator in the activation of macrophages and possibly microglia. Moreover, the risk and pathogenesis of several diseases are correlated with the expression of AIF1. Therefore, AIF1 could be a novel biomarker for prognosis and response to treatment. Additionally, since AIF1 is an intracellular signaling molecule, specific inhibitors that target AIF1 or its expression should be synthesized and evaluated for the treatment of inflammatory diseases, especially those in which macrophages are activated and accelerate pathogenesis.

## Data Availability

Not applicable.
